# Score Images as a Modality: Enhancing Symbolic Music Understanding through Large-Scale Multimodal Pre-Training

**DOI:** 10.3390/s24155017

**Published:** 2024-08-02

**Authors:** Yang Qin, Huiming Xie, Shuxue Ding, Yujie Li, Benying Tan, Mingchuan Ye

**Affiliations:** 1 School of Artificial Intelligence, Guangxi Colleges and Universities Key Laboratory of AI Algorithm Engineering, Guilin University of Electronic Technology, Guilin 541004, China; qinyang@guet.edu.cn (Y.Q.); yujieli@guet.edu.cn (Y.L.); by-tan@guet.edu.cn (B.T.); 2 Engineering Comprehensive Training Center, Guilin University of Aerospace Technology, Guilin 541004, China; xhm@guat.edu.cn; 3 Cloud Computing & Big Data Center, Gongcheng Management Consulting Co., Ltd., Guangzhou 510630, China; allenqy@126.com

**Keywords:** music understanding, transformer, score images, large-scale pre-training

## Abstract

Symbolic music understanding is a critical challenge in artificial intelligence. While traditional symbolic music representations like MIDI capture essential musical elements, they often lack the nuanced expression in music scores. Leveraging the advancements in multimodal pre-training, particularly in visual-language pre-training, we propose a groundbreaking approach: the Score Images as a Modality (SIM) model. This model integrates music score images alongside MIDI data for enhanced symbolic music understanding. We also introduce novel pre-training tasks, including masked bar-attribute modeling and score-MIDI matching. These tasks enable the SIM model to capture music structures and align visual and symbolic representations effectively. Additionally, we present a meticulously curated dataset of matched score images and MIDI representations optimized for training the SIM model. Through experimental validation, we demonstrate the efficacy of our approach in advancing symbolic music understanding.

## 1. Introduction

Music understanding poses a fundamental challenge within the realm of artificial intelligence. A deeper comprehension of melody, rhythm, and music structure not only benefits music information retrieval [1] but also aids in music generation [2,3]. In the domain of machine learning, music has been represented using symbolic notation [4], such as the Musical Instrument Digital Interface (MIDI), which encapsulates crucial musical elements like pitch, duration, and intensity. The advent of Transformer-based [5] pre-training language models [6,7] has revolutionized the field of natural language processing. Similarly, the application of pre-training techniques to MIDI-like symbolic music understanding has demonstrated promising outcomes, mirroring the progress achieved in language-related tasks [8,9,10,11].

However, it is essential to acknowledge that the process of human music creation typically involves composing music scores followed by performance, much like how text is composed and then articulated. Music scores, as highly symbolic and abstract visual expressions, serve as tangible representations of musical ideas, capturing nuances and intricacies that surpass the limitations of MIDI representations. Consequently, certain studies have explored music score images through pre-training methods [12,13,14]. While both MIDI-like representations and score images hold potential significance, current pre-training research has primarily focused on either MIDI or score images individually.

Recently, the domain of multimodal pre-training, particularly visual-language pre-training [15,16,17,18], has experienced significant advancements. Drawing inspiration from this progress and recognizing the symbiotic relationship between scores and MIDI, akin to that between images and text, we propose an innovative large-scale symbolic music pre-training model that regards Score Images as a Modality (SIM) within a multimodal pre-training framework to enhance music-understanding tasks. By integrating music score images with MIDI representations and employing tasks such as masked bar-attribute modeling and score-MIDI matching, our SIM model captures the structural complexity of music and aligns visual and symbolic representations. This fusion of modalities transcends the limitations of single-modal methods, offering a more holistic understanding of music. We also curate a specialized dual-modality dataset to optimize SIM model training, highlighting the significance of incorporating score images in multimodal pre-training. Looking ahead, we aim to explore additional pre-training tasks to guide controllable music generation with pre-trained models, contributing to the broader pursuit of innovation in AI and the development of models that can deeply appreciate and recreate the artistry of music.

The main contributions of this work are outlined as follows:

- Score Images as a Modality. We propose the SIM model, which integrates music score images alongside MIDI data in multimodal pre-training. To our knowledge, we are the first to utilize score images as a distinct modality, thereby enriching the model’s comprehension of symbolic music.
- Novel Pre-training Tasks. We introduce the masked bar-attribute modeling and score-MIDI matching pre-training tasks to enable the SIM model to capture music structures and effectively align visual and symbolic representations. This enhances the model’s ability to interpret diverse musical content.
- Specialized Dataset Creation. We meticulously compile a comprehensive dataset comprising matched pairs of score images and MIDI representations. This dataset is specifically designed to train the SIM model effectively. It is carefully crafted to optimize the training process and highlight the significance of incorporating score images as a modality in multimodal pre-training, thereby enhancing the understanding of symbolic music.

## 2. Related Works

The intersection of artificial intelligence and music understanding represents a significant endeavor to decode the intricate structures and expressive qualities of music, akin to language comprehension and generation. Symbolic music understanding has particularly benefited from methodologies in natural language processing (NLP), with early approaches employing word2vec to generate embeddings for musical notes within a contextual framework [19,20]. Despite their initial promise, these methods were constrained by their focus on smaller neural networks and limited token inputs [19,21].

The emergence of deep pre-trained models, such as BERT, has revolutionized the field by enabling the processing of extended sequences and capturing richer contextual information [6,7,22,23]. In NLP, the efficacy of these models is heavily influenced by masking strategies, with BERT and its variants utilizing random token masking to enhance predictive capabilities [6,24,25,26]. However, adapting these strategies to symbolic music is non-trivial due to the unique structural and informational attributes of music data.

Recent advancements have witnessed MusicBERT and MidiBERT-Piano pushing the boundaries of pre-training techniques in symbolic music understanding [8,9]. These models have demonstrated the potential of large-scale pre-training and tailored masking strategies to enhance learning from musical data. Additionally, Shen et al. [11] contribute a detailed exploration of pre-training strategies, proposing quad-attribute masking and key prediction tasks to improve knowledge extraction from symbolic music.

The visual representation of music scores presents a rich source of information that surpasses MIDI’s capabilities, capturing the subtleties of musical expression. Efforts to integrate music score images with pre-training methods have begun to unlock new potentials for music-understanding tasks, including score following and style identification [12]. An important contribution in this area is the dataset introduced by Lin et al., which provides a comprehensive resource for music score modeling and generation [27]. This dataset, along with others, has facilitated the development of models that can process and interpret the visual aspects of musical scores, offering a more nuanced understanding of musical compositions. The amalgamation of pre-trained models with score images opens novel research pathways, complementing quantitative MIDI data with the qualitative insights from visual representations [13,14].

While foundational, existing studies have predominantly concentrated on single modalities—MIDI or score images. The synergistic integration of both within a unified pre-training framework represents relatively uncharted territory. This research pioneers a novel approach, Score Images as a Modality (SIM), within a multimodal pre-training paradigm for symbolic music understanding. It introduces masked bar-attribute modeling and score-MIDI matching tasks, designed to capture music’s structural complexity and align visual and symbolic representations. To facilitate the SIM model training, we have meticulously compiled a specialized dataset that integrates score images with MIDI representations. This dual-modality dataset not only optimizes the training process but also underscores the importance of incorporating visual modalities into multimodal pre-training frameworks.

## 3. The Proposed Method

**Figure 1:**
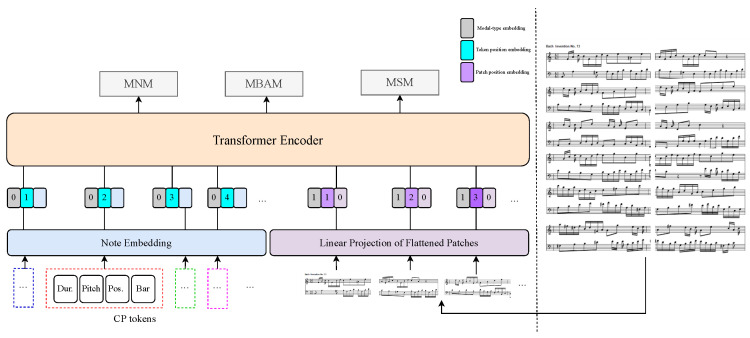
The overall architecture of the proposed SIM model.

As shown in Figure 1, the entire architecture of the proposed SIM is a Transformer-encoder-based framework. The SIM model employs Compound Word-based (CP-based) MIDI music representation for symbolic music input. To suit the patch-based processing paradigm of Vision Transformer [28], we customized the image process for music score images. Specifically, we segmented the score images into patches based on music bars, resulting in an arrangement of 6 × 2 patches (as illustrated on the right side of Figure 1).

### 3.1. MIDI-Score Pre-Training Dataset

In constructing our MIDI-Score Pre-training Dataset (MSD), our primary objective was to integrate diverse musical styles and complexities from multiple public-domain piano MIDI datasets: Pop1K7 [29], MAESTRO [30], GiantMIDI-Piano [31], and Lakh-MIDI [32]. These datasets were selected for their prominence and the breadth of musical information they provide, as detailed in Table 1.

To ensure a harmonious integration of these varied datasets, we implemented a rigorous harmonization process. Initially, we conducted an in-depth analysis to categorize the musical pieces based on their stylistic and complexity-related attributes. This classification facilitated a balanced representation of different musical styles and complexity levels within our dataset:

- Stylistic harmonization. Recognizing the diversity in musical styles, we employed a stratified sampling approach during the dataset assembly. This method ensured proportional representation of various styles, reflecting the eclectic nature of the source datasets. Additionally, stylistic metadata were utilized to annotate each piece in our dataset, allowing for targeted sampling and analysis based on style.
- Complexity harmonization. Addressing the complexity, we developed a normalization algorithm that adjusts for differences in note density, pitch range, and rhythmic variety. This algorithm standardizes the representation of musical complexity, ensuring that pieces of varying difficulty levels are equitably represented and can be effectively processed by our model.
- Transformation to score images. To create a consistent representation for multi-modal pre-training, we transformed the MIDI data into score images using a uniform conversion process. Each MIDI composition was converted into a single score image, encapsulating only the initial 12 bars of music (less than 12 bars are converted). This decision was made to maintain computational feasibility while capturing the essence of each piece. The conversion tool utilized is (http://midisheetmusic.com/index.html) (accessed on 31 July 2024), and we ensured that the process was applied consistently across all datasets.
- Dataset Standardization: To manage computational complexity and ensure uniformity, we implemented a standardization protocol that includes cleansing and deduplication. This process was modeled after the procedures used in MusicBERT [8], applied to both MSD-small and MSD-large subsets. MSD-small, derived from Pop1K7, was subjected to the same rigorous preprocessing as described for MusicBERT, yielding 1589 songs and 1589 MIDI-Score pairs for base pre-training (i.e., MSD-small is used for the base pre-training dataset). MSD-large, an amalgamation of Pop1K7, MAESTRO, GiantMIDI-Piano, and Lakh-MIDI datasets, was processed similarly, resulting in a substantial dataset comprising 159,907 songs and 159,907 MIDI-Score pairs for large-scale pre-training (i.e., MSD-large is used for the large-scale pre-training dataset).

### 3.2. MIDI-Symbolized Representation

A robust representation of MIDI-symbolized music is necessary for the SIM model to capture musical information effectively. MIDI music can be considered as a sequence of “tokens” or musical events, similar to text. However, unlike text, music incorporates note durations and the ability to play multiple notes simultaneously, necessitating the presence of markers for note pitch, duration, and temporal grid placement. Various symbolic music representations have been explored in the past, such as REMI [33], Compound Word (CP) [29], and OctupleMIDI [8]. In this study, the dataset employed for pre-training comprises piano MIDI and corresponding scores. The investigative focus renders a multi-instrumental representation unnecessary.

**Figure 2:**
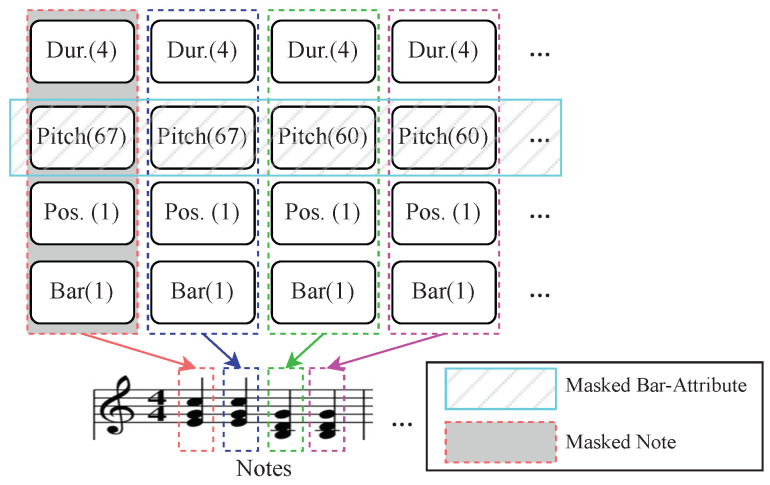
The simplified CP symbolic music representation of a sample piece, with different masking strategies.

Consequently, we adopt the CP token-based approach where each musical event is represented as a token with multiple attributes. A simplified CP token represented each musical note and its four attributes: bar, position, pitch, and duration. As shown in Figure 1 and Figure 2, in musical notation, the term “bar” denotes an indicator of whether a note is the first note within a bar. “position (pos.)” refers to the relative position of the note within the current bar, quantized from 0 to 15. The “pitch” value of the note ranges from 0 to 127, representing the specific musical pitch. Additionally, the “duration (dur.)” value of the note ranges from 1 to 16, indicating the length or duration of the note. To convert the CP tokens in each sequence step into the input of the transformer encoder [5], we concatenate these four attributes and utilize a linear layer to transform them into a unified vector. Let *t* denote a CP token, encapsulating a set of attributes A(t), defined as follows:

(1) A(t)={bar,position,pitch,duration},

The amalgamation of these attributes into a singular vector e(t) is facilitated through a linear transformation, which is mathematically expressed as:

(2) e(t)=W·concat(A(t))+b,

where *W* is the weight matrix and *b* is the bias of the linear layer, which are parameters to be learned. This process is pivotal, as it allows the transformer encoder to process the musical data in a manner that captures the intricate relationships and temporal dynamics inherent in the musical composition. A masking strategy is implemented for CP tokens throughout the pre-training process, as elaborated in Section 3.5.

### 3.3. Score Image Representation

In the realm of music understanding, the role of score image representation is pivotal, as highlighted in our methodology. Our approach leverages the Vision Transformer (ViT) architecture, specifically the ViT-B/16 variant, for encoding score images [28]. This model, initialized with weights pre-trained on the ImageNet-1k dataset, facilitates the transformation of score images into a rich set of embeddings that encapsulate the holistic context of the visual data.

As delineated in Figure 1, our encoding strategy incorporates a meticulous patching mechanism, segmenting each score image into a 12-patch grid (arranged in a 6×2 configuration) prior to processing. This partitioning strategy was the outcome of a deliberate and methodical evaluation of various partitioning schemes. The selection of a 12-patch division resonates with the musical construct of bars, a fundamental unit in Western musical notation that delineates a temporal segment within a composition. Let *I* be a score image, segmented into *N* patches of size P×P. Each patch $p_{i}$ is a discrete segment of the score, representing a musical bar. Each patch $p_{i}$ is represented by utilizing a pre-trained Vision Transformer (ViT) model, yielding a set of vector:

(3) $$e\left(p\right)=\{e\left(p_{1}\right),e\left(p_{2}\right),\ldots ,e\left(p_{N}\right)\},$$

where $e(p_{i})$ is the representation vector for the *i*-th patch, *N* is determined by the patch resolution and the total number of patches. These vectors encapsulate the visual and structural information of the respective patches.

Adopting a 12-bar segment as the basis for our patching strategy ensures that our model captures a musically significant temporal extent, which is congruent with prevalent phrasing patterns in musical compositions. This structure is not only ubiquitous across diverse musical genres but also provides a balanced perspective on the musical phrase, enabling the model to process a segment that is both musically coherent and expressive. Our ablation studies, which explored alternative partitioning schemes, have demonstrated that the 12-bar segmentation strikes an optimal balance between computational expediency and the preservation of nuanced musical elements essential for precise model inference.

The strategic segmentation into 12 patches, each representative of a bar, significantly enhances the robustness and contextual richness of the musical score image representation. This approach is particularly advantageous for the ViT-B/16 model, as it promotes a more profound comprehension of the score’s temporal dynamics and sequential progression.

Recognizing the variability in the length of musical compositions, our model incorporates a rolling window technique to handle compositions that exceed the 12-bar threshold. This technique processes the score in overlapping 12-bar segments, ensuring that the model’s interpretative scope is not limited to the initial 12 bars but is instead extended across the entire compositional expanse.

### 3.4. Representation Embedding and Integration Processing

This section will introduce the theoretical foundation and mathematical derivation of the SIM model depicted in Figure 1. The architecture of SIM is succinct yet powerful, embodying a single-stream approach that streamlines the processing of both visual and symbolic musical data. The model deviates from conventional practices by employing a pre-trained Vision Transformer (ViT) to initialize the interaction transformer weights, thereby leveraging the transformer’s inherent ability to process visual features without the need for a separate visual embedder.

The CP token-based musical representation vector $e\left(t\right)\in R^{L\times |V|}$ is embedded into $\bar{e(t)}\in R^{L\times H}$ using a word embedding matrix $T\in R^{|V|\times H}$ and a position embedding matrix $T_{\mathrm{pos}}\in R^{(L+1)\times H}$:

(4) $$\bar{e(t)}=\left[e\left(t_{1}\right)T;\cdots ;e\left(t_{L}\right)T\right]+T_{\mathrm{pos}},$$

For the visual component, the music score images *I*, represented as $I\in R^{C\times H\times W}$, are segmented into patches aligned with musical bars, resulting in a flattened 6×2 patch representation vector $e\left(p\right)\in R^{N\times (P^{2}\cdot C)}$, where (P,P) denotes the patch resolution and $N=\frac{H\times W}{P^{2}}$. Subsequently, a linear projection $V\in R^{(P^{2}\cdot C)\times H}$ and a position embedding $V_{\mathrm{pos}}\in R^{(N+1)\times H}$ are applied, embedding e(p) into $\bar{e(p)}$:

(5) $$\bar{e(p)}=\left[e\left(p_{1}\right)V;\cdots ;e\left(p_{N}\right)V\right]+V_{\mathrm{pos}},$$

The embeddings $\bar{e(t)}$ and $\bar{e(p)}$ are enhanced by summing them with their respective modal-type embedding vectors $t_{\mathrm{type}}$ and $v_{\mathrm{type}}$, followed by concatenation into a combined sequence $z^{0}$:

(6) $$z^{0}=\left[\bar{e(t)}+t^{\mathrm{type}\;};\bar{e(p)}+v^{\mathrm{type}\;}\right],$$

This integrated sequence sets the stage for the model to process both the visual and symbolic aspects of the musical input in tandem. The contextualized vector *z* is iteratively updated through a series of D-depth transformer layers, as defined by the equations:

(7) $${\hat{z}}^{d}=\mathrm{MSA}\left(\mathrm{LN}\left(z^{d-1}\right)\right)+z^{d-1},\;d=1\ldots D,\;$$

(8) $$z^{d}=\mathrm{MLP}\left(\mathrm{LN}\left({\hat{z}}^{d}\right)\right)+{\hat{z}}^{d},\;d=1\ldots D,\;$$

where MSA denotes the multi-headed self-attention mechanism, LN represents layer normalization, and MLP signifies the multi-layer perceptron within each transformer block.

The final contextualized sequence $z^{D}$ is processed to obtain a pooled representation *m* of the entire multimodal input. This is achieved by applying a linear projection $W_{\mathrm{pool}}\in R^{H\times H}$ followed by a hyperbolic tangent activation to the first index of $z^{D}$, as expressed in the equation:

(9) $$m=\tanh(z_{0}^{D}W_{\mathrm{pool}})$$

The SIM pooled representation encapsulates the rich and contextually enriched information extracted from both visual score images and symbolic MIDI data, thereby affording a comprehensive understanding of the musical input.

### 3.5. MIDI-Score Pre-Training Strategies

We referred to the objectives of visual-language pre-training [15,18] and developed three music multi-modal pre-training tasks for our model: masked note modeling, masked quad-attribute modeling, and MIDI-score matching.

Masked Note Modeling (MNM). As shown in Figure 2, taking inspiration from the masked strategy employed in BERT [6] and MusicBERT [8], we adopt a similar approach wherein 15% of the CP tokens are randomly masked. The task assigned to our SIM model involves predicting (reconstructing) the masked tokens, focusing on minimizing the cross-entropy loss. In alignment with BERT conventions, within the subset of masked tokens, [MASK] tokens replace 80%, 10% are substituted with randomly selected tokens, and the remaining 10% are left unchanged. The masking strategy is based on the idea that the model should be able to infer missing information based on the surrounding context. This is analogous to predicting a word in a sentence based on the surrounding words. Mathematically, the loss function for MNM can be expressed as:

(10) $$L_{\mathrm{MNM}}=-\sum_{i}y_{i}\log\left(p_{i}\right)$$

where $y_{i}$ is the true token and $p_{i}$ is the predicted token by the model.

However, recognizing the potential information leakage caused by the MNM strategy within the unique context of music, hindering the model’s ability to learn contextual representations effectively, we introduce a masked quad-attribute modeling strategy [11] to address this limitation.

Masked Bar-Attribute Modeling (MBAM). Inspired by the approach of quad-attribute masking [11], we introduce the MBAM method, which aims to mitigate information leakage from notes to bars. In the context of music songs, which consist of multiple bars, these bars can be considered highly interconnected. CP tokens encompass note pitch, duration, and temporal grid placement. Moreover, CP tokens can be easily inferred from the adjacent tokens within the same bar. Combining these attributes within a bar provides a more comprehensive global perspective. Therefore, we employ the MQAM technique, wherein each note attribute and its adjacent notes’ attribute within a bar is masked. MQAM allows us to harness a holistic range of musical information, from individual notes to bars.

As delineated in Figure 2, the MBAM approach randomly selects 15% of the tokens corresponding to the music notes. Subsequently, one attribute (bar, position, pitch, or duration) is randomly hidden for each selected note and its adjacent tokens within the same bar. For each masked attribute, there exist three alternative options: (1) in 80% of the cases, it is substituted with a [MASK] token; (2) in 10% of the cases, it is replaced with a randomly selected token from the dictionary; (3) in the remaining 10% of the cases, it remains unchanged. MBAM is based on the principle that musical notes within a bar are interdependent. By masking multiple attributes within a bar, the model is forced to consider the global structure of the music. The loss function for MBAM can be derived from the cross-entropy loss and adjusted for multiple attributes:

(11) $$L_{\mathrm{MBAM}}=-\sum_{j}\sum_{i}y_{ij}\log\left(p_{ij}\right)$$

where $y_{ij}$ is the true attribute of the *i*-th note in the *j*-th bar, and $p_{ij}$ is the predicted attribute.

MIDI-Score Matching (MSM). We aim to fully exploit the self-constructed dataset containing pairs of MIDI and score, and drawing inspiration from tasks of image-text matching [15,18], we propose the MSM task. In the MSM task, the SIM model is presented with a batch of MIDI-score pairs that are either matched or mismatched, and the objective is to determine the correspondence between each MIDI and score image. To introduce variability, we randomly substitute the aligned image with a different image, with a probability of 0.5. The joint representation of the MIDI-score pair is obtained by utilizing the output embedding of the [CLS] token from the transformer encoder. A fully connected (FC) layer followed by a softmax function is appended to predict a two-class probability. The MSM loss is computed by applying the negative log-likelihood loss. This approach aims to enhance the model’s ability to associate corresponding MIDI and score images within the context of our multimodal dataset. MSM is based on the concept of image-text matching, where the model learns to associate textual descriptions with corresponding images. In the context of music, this translates to associating MIDI data with score images. The loss function for MSM is based on the negative log-likelihood loss:

(12) $$L_{\mathrm{MSM}}=-\sum_{k}\log\left(p_{\mathrm{match},k}\right)$$

where $p_{\mathrm{match},k}$ is the predicted probability that the *k*-th MIDI-score pair is a match.

## 4. Experiments

### 4.1. Experiments Setup

Pre-training setup. In our study, we developed two SIM versions. The first version, the SIM_*base*_ model, is pre-trained on the base MSD-small dataset (refer to Table 1 for more information). This version aims to provide a fair comparison with previous works on music understanding, such as MidiBERT [9]. The second version, the SIM_*large*_ model, is pre-trained on the large-scale MSD-large dataset (refer to Table 1 for more information). This version aims to push the state-of-the-art results and demonstrate the scalability of MusicBERT [8].

Downstream tasks. We adopt six music-understanding tasks to compare the efficacy of our proposed SIM model with previous work: two note-level tasks (i.e., melody extraction and velocity prediction), two phrase-level tasks (i.e., melody completion and accompaniment suggestion), and two song-level tasks (i.e., composer and emotion classification).

Implementation details. In our implementation, the models SIM_*base*_ and SIM_*large*_ undergo pre-training on a computational infrastructure comprising 4 NVIDIA V100 GPUs over 11 days. The pre-training regimen consists of 146,000 steps, employing a batch size of 64 sequences, each with a maximum token length of 512. The transformer encoder architecture incorporates 12 multi-head self-attention layers. A preemptive halting mechanism is employed to mitigate the risk of over-fitting, terminating the training process in instances where there is no discernible reduction in the loss value over 30 consecutive epochs. The optimization uses the AdamW optimizer [34], with a learning rate set at 3 × $10^{-5}$ and a weight decay rate of 0.01. These parameters collectively contribute to the stability and efficiency of the training process.

Fine-tuning setup. To fine-tune SIM_*base*_ and SIM_*large*_, we enhance the architecture by modifying the last few layers differently. For note-level and phrase-level understanding tasks, we input the hidden vectors into a stack of dense layers, a ReLU activation layer, and a final dense layer for output classification, incorporating a 10% dropout probability. For song-level understanding tasks, we utilize an attention-based weighting average mechanism to transform the sequence of 512 hidden vectors for an input sequence into a single vector before passing it to the classifier layer, which consists of two dense layers. The parameters initialized by pre-training will be employed, and each task will undergo fine-tuning for 10 epochs. Other details are the same as in the aforementioned pre-training setup.

### 4.2. Note-Level Understanding Tasks

Note-level tasks primarily assess the model’s ability to understand musical notes. The melody extraction task entails the classification of each musical note into three categories: melody, bridge, or accompaniment. We fine-tune our pre-trained SIM models for this task using the POP909 dataset [35]. The velocity prediction task entails classifying each musical note into six velocity categories: pp (0–31), p (32–47), mp (48–63), mf (64–79), f (80–95), and ff (96–127). Our approach also utilizes the POP909 [35] dataset to fine-tune our pre-trained SIM models specific to the velocity prediction task.

We conduct a comparative analysis of SIM with two baseline models in symbolic music understanding: RNN [36] and MidiBERT [9]. The evaluation metric chosen for these note-level tasks is classification accuracy (ACC), assessing the model’s proficiency in accurately categorizing each note. As shown in Table 2, SIM_*large*_ demonstrates a notable improvement, achieving an increased accuracy of 2.69% for melody extraction and 5.12% for velocity prediction when compared to the baseline models. SIM_*base*_ surpasses the performance of RNN and MidiBERT on both melody extraction and velocity prediction tasks using CP token representation (as shown in Figure 2) on the same-scale pre-training dataset. SIM_*base*_ consistently outperformed both RNN and MidiBERT on these tasks, highlighting its superiority in understanding melodic nuances and predicting velocity when leveraging music score images during pre-training on the same scale dataset. Moreover, SIM_*large*_, benefiting from a larger corpus and the inclusion of music score images, demonstrated superior results. This underscores the effectiveness of large-scale multimodal pre-training in enhancing performance in note-level understanding tasks.

### 4.3. Song-Level Understanding Tasks

Song-level tasks evaluate the model’s aptitude for understanding the entirety of a musical sequence. The composer classification task focuses on identifying the composer from the given input token sequence. For fine-tuning our pre-trained SIM models for this task, we employ the Pianist8 [9] dataset, comprising over 400 tracks from eight different composers, each contributing unique musical styles. Similarly, the emotion classification task centers on categorizing the emotion conveyed in the input token sequence. To accomplish this, our pre-trained SIM models are fine-tuned using the EMOPIA [37] dataset, which includes 1087 pieces categorized into four distinct emotion categories.

In our comparative analysis, we benchmarked the SIM model against two baseline models, RNN [36] and MidiBERT [9]. The evaluation metric for song-level tasks is classification accuracy (ACC), gauging the model’s proficiency in accurately categorizing each song. As depicted in Table 2, SIM_*large*_ achieved significant accuracy improvements, with a 9.62% enhancement for composer classification and a notable 14.73% improvement for emotion classification. Notably, SIM_*base*_ exhibited equivalent performance advantages on both tasks when utilizing pre-trained data of the same scale as RNN and MidiBERT, emphasizing its superiority in understanding composer and emotion when leveraging music score images. Furthermore, SIM_*large*_, equipped with a larger pre-training corpus and incorporating music score images, demonstrated superior results. This emphasizes the effectiveness of large-scale multimodal pre-training in enhancing performance in song-level understanding tasks.

### 4.4. Phrase-Level Understanding Tasks

Phrase-level tasks are designed to assess the model’s capability of understanding musical phrases within a given composition. The melody completion task is to locate the most suitable consecutive phrase within a provided set of candidates for a given melodic phrase. Similarly, the accompaniment suggestion task is to identify the most relevant accompaniment phrase from a given set of harmonic phrase candidates for a given melodic phrase. For these two prase-level tasks, we followed the experimental setup of Liang et al. [21] to fine-tune the SIM models.

We employ the mean average precision (MAP) and HITS@k (where k = 1, 5, denoting the proportion of correctly selected phrases among the top k candidates) as evaluation criteria, thereby conducting comparisons with PiRhDy [21], Tonnetz [38], and MusicBERT [8]. As shown in Table 3, SIM_*base*_ demonstrated comparable performance advantages on both tasks when utilizing pre-trained data of the same scale as PiRhDy and MusicBERT. This underscores SIM_*base*_’s superiority in understanding musical phrases, particularly when leveraging additional information from music score images. Moreover, SIM_*large*_, benefiting from a larger pre-training corpus and combining music score images, showcased superior results. This underscores the effectiveness of large-scale multimodal pre-training in enhancing performance in phrase-level understanding tasks.

### 4.5. Ablation Study

To thoroughly assess the individual contributions of SIM’s key components, we conducted an extensive ablation study aimed at evaluating the impact of each element on understanding downstream tasks. The components under investigation include score images as a modality, CP token representation, pre-training strategies, the patches division strategy of score images into patches, and the effects of different visual encoders. We conducted experiments on SIM_*base*_, fine-tuning it for melody extraction, velocity prediction, composer classification, and emotion classification tasks. Our ablation study was designed to systematically isolate and quantify the influence of each component.

Impact of score image modality. Initially, we compared the pre-training performance with and without score images (SI). When score images were not used, only the Masked Note Modeling (MNM) pre-training objective was employed. The results underscored the significant contribution of score images to the model’s overall performance.

Pre-training strategy evaluation. We evaluated the effects of Masked Bar-Attribute Modeling (MBAM) and MIDI-Score Matching (MSM) pre-training strategies by comparing models trained with and without these strategies. Both MBAM and MSM were found to significantly enhance the model’s ability to capture music structures and align visual and symbolic representations.

Token representation comparison. We compared SIM’s CP token representation with the REMI representation, without using the MBAM pre-training objective. The CP token representation consistently outperformed the REMI representation across various tasks, highlighting the importance of an effective token representation for music applications.

Patches division strategy assessment. Recognizing the importance of the division strategy, we further conducted experiments to evaluate the impact of different bar-based patches division strategies on the model’s performance. Alongside our standard 12-bar patches, we tested 8-bar (8-patch division) and 16-bar (8-patch division) strategies.

As shown in Table 4, we observed a dramatic drop in performance when score images were not used for pre-training (row 1). Additionally, our proposed MBAM and MSM pre-training objectives effectively boosted results across all downstream tasks (rows 2 and 3). Furthermore, CP token representation consistently outperformed REMI representation across various tasks, underscoring the importance of token representation for music applications (row 4). The 12-patch division demonstrated the most effective balance, aligning with common phrasing in musical compositions and providing a comprehensive view of the musical structure. Comparatively, the 8-bar division (row 5) resulted in a loss of contextual information, while the 16-bar division (row 6) led to increased computational demand without corresponding performance benefits. Our experiments indicated that the 12-patch division strategy provided the best performance across a range of music-understanding tasks.

Effects of different visual encoders for the SIM_*base*_ model on the testing melody extraction tasks. We have expanded our ablation study to include a detailed numerical analysis of the impact of different visual encoders [28]—specifically, ViT-S/16, ViT-L/16, and ViT-H/16—on model accuracy and computational efficiency. All experiments were conducted under identical conditions, using the same 4 NVIDIA V100 GPUs NVIDIA, Santa Clara, CA, USA, ensuring that variations in performance can be attributed solely to the encoders’ architectural differences. We systematically assessed the performance of each Vision Transformer model variant on the melody extraction task, a critical component of music understanding. Accuracy was used as the primary metric to evaluate performance, and computational time was measured for processing a standardized batch of score images under the same hardware conditions. As shown in Table 5, the ViT-B/16 model offers the best balance of high accuracy and computational efficiency for the SIM model. The results provide clear guidance for model architecture selection, emphasizing the importance of considering both performance metrics and computational efficiency, especially in resource-intensive tasks such as music understanding from score images.

## 5. Conclusions

Our work presents the SIM (Score Images as a Modality) model, a pioneering approach in the realm of multimodal pre-training for music understanding. By harnessing the synergistic power of MIDI data and music score images, SIM transcends traditional symbolic music interpretation through its innovative pre-training tasks and a meticulously curated dataset. The integration of score images as a distinct modality within our framework is a significant step forward in the field of artificial intelligence, offering new pathways for the enhanced comprehension of music.

The SIM model’s success in symbolic music understanding is a testament to the potential of multimodal pre-training. It demonstrates that by bridging the gap between notated music and audio sounds, we can achieve deeper insights into the structural and expressive qualities of music. Our model’s performance across a variety of music-understanding tasks sets a new benchmark for the field.

Looking ahead, there are several promising directions for future research. First, we aim to explore additional pre-training tasks that could further enhance the model’s ability to capture the nuances of musical expression. Second, we are interested in investigating how the SIM model could be adapted to handle different musical genres and notational styles, thereby broadening its applicability. Third, we will examine methods to improve the computational efficiency of our model, ensuring that it can be scaled to accommodate larger datasets and more complex musical compositions.

Furthermore, we are excited about the prospect of integrating the SIM model with other AI technologies to enable advanced applications in music generation, performance, and education. By doing so, we hope to contribute not only to the academic community but also to the broader ecosystem of music creation and appreciation.

In conclusion, the SIM model represents a significant advancement in the intersection of artificial intelligence and music understanding. With its novel approach to multimodal pre-training, our work lays the groundwork for future innovations that will continue to push the boundaries of what is possible in this exciting domain.

## Figures and Tables

**Table 1:** The sizes of different music datasets.

Datasets	Usage	# Songs	# MIDI-Score Pairs
Pop1K7	pre-training (base)	1747	×
MAESTRO	pre-training (large)	1184	×
GiantMIDI-Piano	pre-training (large)	10,854	×
Lakh-MIDI	pre-training (large)	176,581	×
MSD-small (Ours)	pre-training (base)	1589	1589
MSD-large (Ours)	pre-training (large)	159,907	159,907

**Table 2:** The testing classification accuracy (in %) of various models for both note-level and song-level understanding downstream tasks, including melody extraction, velocity prediction, composer classification, and emotion classification.

Models	Melody	Velocity	Composer	Emotion
RNN	88.66	43.77	60.32	54.13
MidiBERT	96.37	51.63	78.57	67.89
SIM_*base*_	98.22	54.42	84.68	78.31
SIM_*large*_	99.06	56.75	88.19	82.62

**Table 3:** Results of various models on the song-level understanding downstream tasks: melody completion, accompaniment suggestion.

Model	Melody Completion			Accompaniment Suggestion		
	MAP	HITS@1	HITS@5	MAP	HITS@1	HITS@5
Tonnetz	0.683	0.545	0.865	0.423	0.101	0.407
PiRhDy	0.971	0.950	0.995	0.567	0.184	0.540
MusicBERT	0.985	0.975	0.997	0.946	0.333	0.857
SIM_*base*_	0.991	0.988	1.000	0.963	0.536	0.912
SIM_*large*_	0.997	0.999	1.000	0.985	0.667	0.977

**Table 4:** Testing classification accuracy (in %) ablation experiments for SIM_*base*_ model on downstream tasks.

Models	Melody	Velocity	Composer	Emotion
SIM_*base*_ w/o SI	88.89	44.87	61.84	58.26
SIM_*base*_ w/o MBAM	96.33	50.62	79.41	72.89
SIM_*base*_ w/o MSM	94.98	48.66	77.36	70.64
SIM_*base*_+REMI	92.92	51.43	77.12	71.53
SIM_*base*_ (8-Patch Division)	96.83	51.62	82.44	77.01
SIM_*base*_ (16-Patch Division)	97.19	52.33	83.38	77.77
SIM_*base*_ (ours)	98.22	54.42	84.68	78.31

**Table 5:** Effects of different visual encoders for the SIM_*base*_ model on the testing melody extraction tasks.

Vision Encoder	Accuracy (Melody Extraction)	Computational Time per Batch (seconds)
ViT-S/16	96.83	0.23
ViT-B/16	98.22	0.48
ViT-L/16	98.28	1.06
ViT-H/16	98.33	1.98

## Data Availability

The original contributions presented in the study are included in the article, further inquiries can be directed to the corresponding author.
